# Asymptotics of commuting $\ell$-tuples in symmetric groups and log-concavity

**DOI:** 10.1007/s40993-024-00562-1

**Published:** 2024-10-03

**Authors:** Kathrin Bringmann, Johann Franke, Bernhard Heim

**Affiliations:** Division of Mathematics, Department of Mathematics and Computer Science, University of Cologne, Weyertal 86-90, 50931 Cologne, Germany

**Keywords:** Generating functions, Log-concavity, Partition numbers, Symmetric group, Primary 05A17, 11P82, Secondary 05A20

## Abstract

Denote by $N_{\ell }(n)$ the number of $\ell$-tuples of elements in the symmetric group $S_n$ with commuting components, normalized by the order of $S_n$. In this paper, we prove asymptotic formulas for $N_\ell (n)$. In addition, general criteria for log-concavity are shown, which can be applied to $N_\ell (n)$ among other examples. Moreover, we obtain a Bessenrodt-Ono type theorem which gives an inequality of the form $c(a)c(b) > c(a+b)$ for certain families of sequences *c*(*n*).

## Introduction and statement of results

In this paper we consider asymptotics for commuting $\ell$-tuples in $S_n$, where $S_n$ denotes the symmetric group for $n,\ell \in \mathbb {N}$. To be more precise, let |*M*| be the cardinality of a set *M* and define

1.1 $$\begin{aligned} C_{\ell ,n}:= \left\{ (\pi _1, \ldots , \pi _{\ell }) \in S_n^{\ell } : \pi _j \pi _k = \pi _k \pi _j \text { for } 1 \le j,k \le \ell \right\} . \end{aligned}$$

The numbers $|C_{\ell ,n}|$ are divisible by $\left| S_n \right|$ and appear as the specialization of the $\ell$th orbifold characteristic to the *n*th symmetric product of a manifold of ordinary Euler characteristic 1 (see [2] for a combinatorial approach and Theorem 2.1 of [11]). In this paper, we prove asymptotics and log-concavity of

1.2 $$\begin{aligned} N_{\ell }(n):= \frac{\left| C_{\ell ,n}\right| }{\left| S_n \right| }. \end{aligned}$$

Bryan and Fulman [11]^1^ proved the following.^2^

### Theorem 1.1

(Bryan and Fulman, see also [2], p. 3) For $\ell \in \mathbb {N},$ we have

$$\begin{aligned} \sum _{n\ge 0} \vert {C}_{\ell ,n}\vert \frac{q^n}{n!} =\prod _{n\ge 1} \bigg ( 1-q^n\bigg )^{- g_{\ell -1}(n)} = \exp \left( \sum _{n\ge 1} g_{\ell }(n) \frac{q^n}{n}\right) , \end{aligned}$$

where^3^$g_{\ell }(n)$ denote the number of subgroups of $\mathbb {Z}^{\ell }$ of index *n*.

Since $g_2(n)=\sigma _1(n)$, where for $m \in \mathbb {N}$, $\sigma _m(n):= \sum _{d \vert n} d^m$, we obtain that $N_{2}(n)=p(n)$, where *p*(*n*) denotes the number of partitions of *n* (for more background, see [4, 24]). Thus, $N_{2}(n)$ equals to the number of conjugacy classes in $S_n$ [14]. Note that $\ell =3$ reveals an interesting connection to topology as highlighted by Britnell (see the introduction of [10]) due to work of Liskovets and Medynkh [19]. Namely $N_{3}(n)$ counts the number of non-equivalent *n*-sheeted coverings of a torus. The study of log-concavity in the enumeration of tuples of of commuting permutations was initiated by Abdesselam [1], where he conjectured the log-concavity in *k* of the numbers $A(\ell ,n,k)$ which count $\ell$-tuples of commuting permutations whose joint action on a set of *n* elements has *k* orbits. Recently, Abdesselam, Neuhauser, and one of the authors [3] proved that $N_{\ell }(n)$ is log-concave for $n\ge 20$ for almost all $\ell$ if and only if $n \equiv 0 \pmod {3}$. Moreover it was shown by Nicolas ([23], Proposition 1) and reproved by DeSalvo and Pak ([13], Theorem 1.1) that $N_2(n)$ is log-concave for $n \ge 26$. In [3] it was conjectured that $N_3(n)$ is also log-concave for $n \ge 22$ (numerically verified there for $n \le 10^5$). Based on numerical experiments they also speculated that for fixed $\ell$, $N_{\ell }(n)$ is log-concave for almost all *n*.

In this paper, using results by Bridges, Brindle, and two of the authors in [8], we prove asymptotic formulas for $N_\ell (n)$ for arbitrary $\ell$, and partially answer the question (5) posed in Sect. 6 there. Note that we are in the situation of multiple poles which is not covered by the classical result of Meinardus [22].

### Theorem 1.2

For $\ell \ge 6$ we have,^4^ as $n \rightarrow \infty ,$

$$\begin{aligned} N_\ell (n) \sim \frac{(\ell -1)!^{\frac{1}{2\ell }} \sqrt{Z_\ell }}{\sqrt{2\pi \ell } n^{\frac{\ell +1}{2 \ell }}} \exp \left( \frac{\ell \Gamma (\ell )^{\frac{1}{\ell }}Z_\ell }{\ell -1} n^{\frac{\ell -1}{\ell }} + \sum _{k=2}^{\ell } A_{\ell ,k} n^{\frac{\ell -k}{\ell }}\right) \left( 1 + \sum _{j\ge 1} \frac{B_{\ell ,j}}{n^{\frac{j}{\ell }}}\right) , \end{aligned}$$

where $Z_\ell := (\zeta (2) \cdot \zeta (3) \cdots \zeta (\ell ))^{\frac{1}{\ell }}$ for certain $A_{\ell ,k}$ and $B_{\ell ,j}$.

### Remarks

1. We give a more explicit description for the constants $A_{\ell ,k}$ in (3.21). Theoretically, the values $B_{\ell ,j}$ can also be calculated explicitly. They result from the calculation methods in [8] by a rather complicated procedure.
2. The cases $\ell \in \{3,4,5\}$ are simpler and of special interest. They are treated separately in Theorems 3.2 and 3.3.

It turns out that asymptotics like the one in Theorem 1.2 are sufficient to prove log-concavity^5^ of sequences. Recall that a sequence $a_n$ is called *log-concave* if $a_n^2\ge a_{n+1}a_{n-1}$. We prove the following general result.

### Theorem 1.3

Assume that *c*(*n*) is a sequence with

$$\begin{aligned} c(n) \sim \frac{C}{n^\kappa } \exp \left( \sum _{\lambda \in \mathcal {S}}A_\lambda n^\lambda \right) \sum _{\mu \in \mathcal {T}} \frac{\beta _\mu }{n^\mu } \quad (n \rightarrow \infty ). \end{aligned}$$

Here $\kappa \in \mathbb {R},$ $\mathcal {S}\subset \mathbb {Q}^+ \cap (0,1)$ is finite, $\mathcal {T}\subset \mathbb {Q}_{0}^+,$ *C*,  $A_\lambda ,$ $\beta _\mu \in \mathbb {R}$ with $\beta _0 = 1$. Let $\lambda ^{*}:= \max \{\lambda \in \mathcal {S}: A_{\lambda } \ne 0\}$ and assume that $A_{\lambda ^*}>0$. Then, for *n* sufficiently large, *c*(*n*) is log-concave.^6^

In particular, from Theorems 1.3 and 1.2, we conclude the following.

### Corollary 1.4

Let^7^$\ell \ge 2$. For *n* sufficiently large $N_{\ell }(n)$ is log-concave.

### Remark

In Corollary 4.2 we give as further examples partitions into *k*-gonal numbers, as well as *n*-dimensional representation numbers of the groups $\mathfrak {su}(3)$ and $\mathfrak {so}(5)$.

In 2016, Bessenrodt and Ono ([6], Theorem 2.1) proved that if $a, b \in \mathbb {N}$ satisfy $a, b > 1$ and $a+b > 8,$ then $p(a)p(b) > p(a+b)$ if $\{a,b\}\not = \{2,7\}$. We also show a Bessenrodt–Ono type theorem for general sequences, which implies Bessenrodt’s and Ono’s result on *p*(*n*) for *a*, *b* sufficiently large.

### Theorem 1.5

Let *c*(*n*) be a sequence satisfying

$$\begin{aligned} c(n) \sim \frac{C}{n^\kappa } \exp \left( \sum _{\lambda \in \mathcal {S}} A_\lambda n^\lambda \right) \end{aligned}$$

with $\kappa \in \mathbb {R},$ $\mathcal {S}\in \mathbb {R}\cap (0,1)$ be finite, $C, A_{\lambda }\in \mathbb {R}$. Let $\lambda ^{*}:=\max \{\lambda \in \mathcal {S}: A_{\lambda }\ne 0\}$ with $A_{\lambda ^*} > 0$. If $a,b\gg 1,$ then

$$\begin{aligned} c(a)c(b) > c(a+b). \end{aligned}$$

Again, we can apply this to the sequences $N_\ell (n)$.

### Corollary 1.6

Let $\ell \ge 2$. We have, for $a,b\gg 1$

$$\begin{aligned} N_{\ell }(a)N_{\ell }(b) > N_{\ell }(a+b). \end{aligned}$$

The paper is organized as follows. In Sect. 2, we recall known results. In Sect. 3 we prove Theorem 1.2. In Sect. 4, we show our main results concerning log-concavity and give some examples. In Sect. 5 we provide a proof of Theorem 1.5. In Sect. 6, we state some open questions.

## Preliminaries

### Previous results on asymptotic expansions

We require some results from [8]. Let $f:\mathbb {N}\rightarrow \mathbb {N}_0$, set $\Lambda :=\mathbb {N}\setminus f^{-1}(\{0\})$, and for $q=e^{-z}$ [$z\in \mathbb {C}$ with $\textrm{Re}(z) > 0$], define

$$\begin{aligned} G_f(z):=\sum _{n\ge 0} p_f(n)q^n := \prod _{n\ge 1} \frac{1}{\left( 1-q^n\right) ^{f(n)}},\quad L_f(s) := \sum _{n\ge 1} \frac{f(n)}{n^s}. \end{aligned}$$

We let $\mathcal {P}$ be the set of poles of

$$L^*_f(s):= \Gamma (s)\zeta (s+1)L_f(s),$$

and for $R>0$ we denote by $\mathcal {P}_R$ the union of the poles of $L_f^*$ greater than $-R$ with $\{0\}$. We require the following key properties of these objects:

1. All poles of $L_f$ are real. Let $\alpha >0$ be the largest pole of $L_f$. There exists $L\in \mathbb {N}$, such that for all primes *p*, we have $|\Lambda \setminus (p\mathbb {N}\cap \Lambda )|\ge L>\frac{\alpha }{2}$.
2. Condition (P2) is attached to $R\in \mathbb {R}^+$. The series $L_f(s)$ converges for some $s\in \mathbb {C}$, has a meromorphic continuation to $\{s\in \mathbb {C}:\textrm{Re}(s)\ge -R\}$, and is holomorphic on the line $\{s\in \mathbb {C}:\textrm{Re}(s)=-R\}$. The function $L_f^*(s)=\Gamma (s)\zeta (s+1)L_f(s)$ has only real poles $0<\alpha :=\gamma _1>\gamma _2>\cdots$ that are all simple, except the possible pole at $s=0$, that may be double.
3. For some $a<\frac{\pi }{2}$, in every strip $\sigma _1\le \sigma \le \sigma _2$ in the domain of holomorphicity of $L_f(s)$, we uniformly have, for $s=\sigma +it$,
  $$\begin{aligned} L_f(s) = O_{\sigma _1, \sigma _2}\left( e^{a|t|}\right) , \quad |t| \rightarrow \infty . \end{aligned}$$

We have the following asymptotic behavior of $p_f(n)$.

#### Theorem 2.1

([8], Theorem 1.4) Assume (P1) for $L\in \mathbb {N},$ (P2) for $R>0,$ and (P3). Then, for some $M,N\in \mathbb {N},$ we have

$$\begin{aligned} p_f(n) = \frac{C}{n^b} \exp \left( A_1n^\frac{\alpha }{\alpha +1}+\sum _{j=2}^M A_jn^{\alpha _j}\right) \left( 1+\sum \limits _{j=2}^N \frac{B_j}{n^{\beta _j}} + O_{L,R}\left( n^{-\min \left\{ \frac{2L-\alpha }{2(\alpha +1)},\frac{R}{\alpha +1}\right\} }\right) \right) . \end{aligned}$$

Here, $0\le \alpha _M<\alpha _{M-1}<\cdots \alpha _2<\alpha _1=\frac{\alpha }{\alpha +1}$ are given by $\mathcal {L}$

$$\begin{aligned} \mathcal {L}:= \frac{1}{\alpha +1}\mathcal {P}_R + \sum _{\mu \in \mathcal {P}_R} \left( \frac{\mu +1}{\alpha +1}-1\right) \mathbb {N}_0. \end{aligned}$$

The exponents $0<\beta _2<\beta _3<\cdots$ are given by $\mathcal {M}+\mathcal {N},$ where $\mathcal {M}$ and $\mathcal {N}$ are defined by

$$\begin{aligned} \mathcal {M}&:= \left\{ \theta = \frac{\alpha }{\alpha +1}n + \sum _{\mu \in \mathcal {P}_R} \left( 1 - \frac{\mu +1}{\alpha +1}\right) n_\mu :n, n_\mu \in \mathbb {N}_0, \theta \in \left[ 0,\frac{R+\alpha }{\alpha +1}\right) \right\} , \\ \mathcal {N}&:= \left\{ \sum _{j=1}^K b_j\theta _j : b_j,K\in \mathbb {N}_0,\theta _j\in (-\mathcal {L})\cap \left( 0,\frac{R}{\alpha +1}\right) \right\} . \end{aligned}$$

The coefficients $A_j$ and $B_j$ can be calculated explicitly; $A_1,$ *C*,  and *b* are defined as

$$\begin{aligned}&A_1 := \left( 1+\frac{1}{\alpha }\right) (\omega _\alpha \Gamma (\alpha +1)\zeta (\alpha +1))^\frac{1}{\alpha +1},\quad C := \frac{e^{L_f'(0)}(\omega _\alpha \Gamma (\alpha +1)\zeta (\alpha +1))^\frac{\frac{1}{2}-L_f(0)}{\alpha +1}}{\sqrt{2\pi (\alpha +1)}},\\&b := \frac{1-L_f(0)+\frac{\alpha }{2}}{\alpha +1}, \end{aligned}$$

where $\omega _\nu := \textrm{Res}_{s=\nu } L_f(s)$. Moreover, if $\alpha$ is the only positive pole of $L_f,$ then we have $M=1$.

The situation of exactly two positive poles was worked out explicitly in Theorem 4.4 of [8].

#### Theorem 2.2

Assume that $f:\mathbb {N}\rightarrow \mathbb {N}_0$ satisfies the conditions of Theorem 2.1 and that $L_f$ has exactly two positive poles $\alpha >\beta ,$ such that $\frac{\lambda +1}{\lambda }\beta <\alpha \le \frac{\lambda }{\lambda -1}\beta$ for some $\lambda \in \mathbb {N}$. Then

$$\begin{aligned} p_f(n)&= \frac{C}{n^b}\exp \left( A_1n^\frac{\alpha }{\alpha +1}+A_2n^\frac{\beta }{\alpha +1}+\sum _{k=3}^{\lambda +1} A_kn^{\frac{(k-1)\beta }{\alpha +1}+\frac{k-2}{\alpha +1}+2-k}\right) \\&\quad \times \left( 1+\sum _{j=2}^{N} \frac{B_j}{n^{\nu _j}} + O_{L,R}\left( n^{-\min \left\{ \frac{2L-\alpha }{2(\alpha +1)},\frac{R}{\alpha +1}\right\} }\right) \right) ,\quad (n\rightarrow \infty ), \end{aligned}$$

with

$$\begin{aligned} A_1 := (\omega _{\alpha }\Gamma (\alpha +1)\zeta (\alpha +1))^\frac{1}{\alpha +1}\left( 1+\frac{1}{\alpha }\right) ,\quad A_2 := \frac{\omega _{\beta }\Gamma (\beta )\zeta (\beta +1)}{(\omega _\alpha \Gamma (\alpha +1)\zeta (\alpha +1))^\frac{\beta }{\alpha +1}}, \end{aligned}$$

and for $$k \ge 3$$

$$\begin{aligned} A_k&:= K_k + \frac{c_1^\frac{1}{\alpha +1}}{\alpha }\sum _{m=1}^\lambda \left( {\begin{array}{c}-\alpha \\ m\end{array}}\right) \sum _{\begin{array}{c} 0\le j_1,\dots ,j_\lambda \le m\\ j_1+\cdots +j_\lambda =m\\ j_1+2j_2+\cdots +\lambda j_\lambda =k-1 \end{array}} \left( {\begin{array}{c}m\\ j_1,j_2,\dots ,j_\lambda \end{array}}\right) \frac{K_2^{j_1}\cdots K_{\lambda +1}^{j_\lambda }}{c_1^\frac{m}{a+1}}\\&\quad + \frac{c_2}{\beta c_1^\frac{\beta }{a+1}}\sum _{m=1}^\lambda \left( {\begin{array}{c}-\beta \\ m\end{array}}\right) \sum _{\begin{array}{c} 0\le j_1,\dots ,j_\lambda \le m\\ j_1+\cdots +j_\lambda =m\\ j_1+2j_2+\cdots +\lambda j_\lambda =k-2 \end{array}} \left( {\begin{array}{c}m\\ j_1,j_2,\dots ,j_\lambda \end{array}}\right) \frac{K_2^{j_1}\cdots K_{\lambda +1}^{j_\lambda }}{c_1^\frac{m}{a+1}}. \end{aligned}$$

Here, ${m \atopwithdelims ()m_1, m_2, \ldots , m_k}:= \frac{m!}{m_1! m_2! \cdots m_k!}$ with $\sum _{j=1}^k m_j = m$ denotes the multinomial coefficient. The $\nu _j$ run through $\mathcal {M}+\mathcal {N},$ the $K_j$ are given in Lemma 4.3 of [8], and $c_1,$ $c_2,$ and $c_3$ are defined by

$$\begin{aligned} c_1 := \omega _{\alpha } \Gamma (\alpha +1)\zeta (\alpha +1), \quad c_2 := \omega _{\beta } \Gamma (\beta +1) \zeta (\beta +1), \quad c_3 := L_f(0). \end{aligned}$$

#### Remark

By Lemma 4.3 of [8], the first values of $K_j$ are given by

$$\begin{aligned}&K_1 = c_1^\frac{1}{\alpha +1}, \quad K_2 = \frac{c_2}{(\alpha +1)c_1^\frac{\beta }{\alpha +1}}, \quad K_3 = \frac{c_2^2(\alpha -2\beta )}{2(\alpha +1)^2c_1^\frac{2\beta +1}{\alpha +1}}, \\&K_4 = \frac{c_2^3\left( 2\alpha ^2-9\alpha \beta -2\alpha +9\beta ^2+3\beta \right) }{6(\alpha +1)^3c_1^\frac{3\beta +2}{\alpha +1}}, \\&K_5 = \frac{c_2^4(6 \alpha ^3-44 \alpha ^2 \beta -15 \alpha ^2+96 \alpha \beta ^2+56 \alpha \beta +6 \alpha -64 \beta ^3-48 \beta ^2-8 \beta )}{24(\alpha +1)^4 c_1^{\frac{4\beta + 3}{\alpha + 1}}}. \end{aligned}$$

#### Remark

As the number of positive poles increases, the situation quickly becomes much more complicated. The focus of this paper is, in particular, the case of three poles.

We also require the behavior of a certain saddle point function. We adopt the notation for the coefficients of asymptotic expansions from [8] and write for a sequence *g*(*n*)

$$\begin{aligned} g(n) = \sum _{j = 1}^{N} \frac{a_{g,j}}{n^{\nu _{j}}} + O_R\left( n^{-R}\right) , \quad (\nu _1< \nu _2< \cdots< \nu _R < R). \end{aligned}$$

#### Proposition 2.3

(Corollary 3.4 of [8]) Let $\Phi _f:= \textrm{Log}(G_f)$ and assume that $f:\mathbb {N}\rightarrow \mathbb {N}_0$ satisfies the conditions of Theorem 2.1. Let $\varrho _n > 0$ solve^8^

$$\begin{aligned} -\Phi '_f(\varrho ) = n. \end{aligned}$$

Then

$$\begin{aligned} \varrho _{n} = \sum _{1 \le j \le N_\varrho } \frac{a_{\varrho ,j}}{n^{\nu _{\varrho ,j}}} + O\left( n^{-\frac{R}{\alpha +1}-1}\right) \end{aligned}$$

with $a_{\varrho ,1}=a_{-\Phi _f',1}^\frac{1}{\alpha +1} = (\omega _\alpha \Gamma (\alpha +1)\zeta (\alpha +1))^{\frac{1}{\alpha +1}}$ and we have

$$\begin{aligned} \{\nu _{\varrho ,j}:1\le j\le N_\varrho \} =\left( \frac{1}{\alpha +1} - \sum _{\begin{array}{c} \mu \in {\mathcal {P}_R} \end{array}}\left( \frac{\mu +1}{\alpha +1} - 1 \right) \mathbb {N}_0\right) \cap \left[ \frac{1}{\alpha + 1},\frac{R}{\alpha + 1} + 1\right) . \end{aligned}$$

The following is required for a more explicit investigation of the involved constants.

#### Lemma 2.4

(Lemma 4.1 of [8]) Let $f:\mathbb {N}\rightarrow \mathbb {N}_0$ satisfy the conditions of Theorem 2.1. Then

$$\begin{aligned} p_f(n) = \frac{e^{n\varrho _{n}}G_f(\varrho _n)}{\sqrt{2\pi }}\left( \sum \limits _{j=1}^M \frac{d_j }{n^{\nu _j}} + O_{L,R}\left( n^{-\min \left\{ \frac{L+1}{\alpha +1}, \frac{R+\alpha }{\alpha +1}+\frac{\alpha +2}{2(\alpha +1)}\right\} }\right) \right) \end{aligned}$$

for some $M\in \mathbb {N},$ $d_1=\frac{1}{\sqrt{\alpha +1}}(\omega _\alpha \Gamma (\alpha +1)\zeta (\alpha +1))^\frac{1}{2(\alpha +1)},$ and $\nu _j$ runs through

$$\begin{aligned} \frac{\alpha +2}{2(\alpha +1)} + \frac{\alpha }{\alpha +1}\mathbb {N}_0 + \left( -\sum _{\mu \in \mathcal {P}_R} \left( \frac{\mu +1}{\alpha +1}-1\right) \mathbb {N}_0\right) \cap \left[ 0,\frac{R+\alpha }{\alpha +1}\right) . \end{aligned}$$

In particular, we have $\nu _1 =\tfrac{\alpha +2}{2(\alpha +1)}$.

### Results from complex analysis

We require the Lagrange inversion formula.

#### Lemma 2.5

(Corollary 11.2 of [12]) Let $\phi :B_r(0)\rightarrow D,$ where for $r \in \mathbb {R}^+$ as usual $B_r(0):= \{ z \in \mathbb {C}:|z| < r\},$ be a holomorphic function such that $\phi (0)=0$ and $\phi '(0)\ne 0,$ with $\phi (z)=:\sum _{n\ge 1}a_nz^n$. Then $\phi$ is locally biholomorphic and its local inverse has a power series expansion $\phi ^{-1}(w)=:\sum _{k\ge 1}b_kw^k,$ with

$$\begin{aligned} b_k = \frac{1}{ka_1^{k}} \sum _{\begin{array}{c} \ell _1,\ell _2, \ell _3 \dots \ge 0 \\ \ell _1 + 2\ell _2 + \cdots = k-1 \end{array}} (-1)^{\ell _1 + \ell _2 +\ell _3+\cdots } \frac{k \cdots (k-1+\ell _1+\ell _2+\cdots )}{\ell _1! \ell _2! \ell _3! \cdots }\left( \frac{a_2}{a_1}\right) ^{\ell _1} \left( \frac{a_3}{a_1}\right) ^{\ell _2}. \end{aligned}$$

We also need the growth behavior of the Riemann zeta function in vertical strips.

#### Lemma 2.6

(Theorem 12.23. on p. 270 of [5]) Let $\sigma _1 \le \sigma _2$ be real. Then there exists a constant $B:= B(\sigma _1, \sigma _2) > 0,$ such that for $|t| \ge 1$ and $\sigma _1 \le \sigma \le \sigma _2,$ we have

$$\begin{aligned} |\zeta (\sigma + it)| \ll _{\sigma _1, \sigma _2} |t|^{B}. \end{aligned}$$

To study the asymptotics of $N_{\ell }(n)$ we first have to investigate the analytic properties of the Dirichlet series attached to the sequence $g_{\ell -1}(n)$ from Theorem 1.1 with $f_{\ell }:=g_{\ell -1}$. Solomon [27] (see also [20], Theorem 15.1, p. 296) proved that, for $\ell \ge 2$,

2.1 $$\begin{aligned} L_{f_{\ell }}(s) = \prod _{k=0}^{\ell -2} \zeta (s- k). \end{aligned}$$

For the following investigations the following result is essential; its proof follows from standard properties of the Riemann zeta function.

#### Proposition 2.7

The function $L_{f_\ell }$ has one pole at $s=1$ for $\ell =2,$ two poles $s\in \{1,2\}$ for $\ell =3,$ and three poles $s\in \{\ell -3, \ell -2,\ell -1\}$ for $\ell \ge 4$.

If $\ell \ge 4,$ then we have for $\nu \in \{ \ell -3, \ell -2, \ell -1 \}$

$$\begin{aligned} \textrm{Res}_{s = \nu } L_{f_\ell }^*(s) = (\nu -1)!\zeta (\nu +1) \prod _{\begin{array}{c} 0\le k\le \ell -2\\ k \not = \nu -1 \end{array}} \zeta (\nu - k) \end{aligned}$$

and $L_{f_\ell }(0) = 0$. Additionally, for $\ell \ge 6$ the function $L^*_{f_\ell }$ only has simple poles in $s \in \{\ell -3, \ell -2, \ell -1\}$. For $\ell \in \{4,5\},$ $L_{f_\ell }^*$ has an additional simple pole in $s=0,$ with residue

$$\begin{aligned} \textrm{Res}_{s=0} L_{f_4}^*(s) = \frac{\zeta '(-2)}{24}, \quad \textrm{Res}_{s=0} L_{f_5}^*(s) = \frac{\zeta '(-2)}{2880}. \end{aligned}$$

## Asymptotic expansions for $N_\ell (n)$

### Exponent sets

For our calculations, we need the following lemma which follows by a direct calculation.

#### Lemma 3.1

For $\ell \ge 3$ and $f = f_\ell ,$ we have, as $R \rightarrow \infty ,$

$$\begin{aligned} \mathcal {L}&= \frac{\ell -1}{\ell } - \frac{1}{\ell } \mathbb {N}_0, \quad \mathcal {M} = \frac{1}{\ell } \mathbb {N}_0, \quad \mathcal {N} = \frac{1}{\ell } \mathbb {N}_0. \end{aligned}$$

### The case $\ell = 2$

This case is the partition function and is classical. In the setting of the present paper we have

$$\begin{aligned} N_2(n) = p(n) = \frac{e^{\pi \sqrt{\frac{2n}{3}}}}{4\sqrt{3}n}\left( 1+\sum _{j=1}^N \frac{B_j}{n^\frac{j}{2}}+O_N\left( n^{-\frac{N+1}{2}}\right) \right) \end{aligned}$$

for certain $B_j$ and this case was treated in [8].

### The case $\ell = 3$

The following theorem gives the asymptotic behavior of $N_{3}(n)$.

#### Theorem 3.2

We have, as $n \rightarrow \infty ,$

$$\begin{aligned} N_{3}(n) \sim \frac{e^{-\frac{\zeta '(-1)}{2} -\frac{\pi ^2}{288 \zeta (3)}}\zeta (3)^{\frac{11}{72}}}{2^{\frac{11}{24}} \cdot 3^{\frac{47}{72}} \cdot \pi ^{\frac{11}{72}} \cdot n^{\frac{47}{72}}} \exp \left( \frac{(3\pi )^{\frac{2}{3}} \zeta (3)^{\frac{1}{3}}}{2} n^{\frac{2}{3}} - \frac{\pi ^{\frac{4}{3}}}{4 \cdot 3^{\frac{2}{3}} \cdot \zeta (3)^{\frac{1}{3}}} n^{\frac{1}{3}} \right) \left( 1 + \sum _{j \ge 1} \frac{B_{3,j}}{n^{\frac{j}{3}}}\right) \end{aligned}$$

for certain numbers $B_{3,j}$.

#### Proof

We have by (2.1) (see [5], p. 231)

3.1 $$\begin{aligned} L_{f_3}(s) = \zeta (s)\zeta (s-1) = \sum _{n \ge 1} \frac{\sigma _1(n)}{n^s}. \end{aligned}$$

Thus the only positive poles of $L_{f_3}(s)$ are $\alpha = 2$ and $\beta =1$. Moreover, as $f_3(n) = \sigma _1(n) \not = 0$ for $n \in \mathbb {N}$, we have $f_3^{-1}(\{0\}) = \emptyset$, and hence we can choose *L* arbitrarily large. We see that (P1) is satisfied. We have $\mathcal {P} = \{1,2\}$ and these poles are simple, so (P2) is satisfied. Note that we may choose *R* arbitrarily large, as $\zeta (s)\zeta (s-1)$ has a meromorphic continuation to the entire complex plane. Applying Lemma 2.6, we have that $L_{f_3}(\sigma +it) \ll e^{a|t|}$ in vertical strips with finite width for arbitrary $a > 0$, so in particular (P3) is satisfied. Thus we can use Theorem 2.2.

First, we note that $\lambda =2$ satisfies $\frac{\lambda +1}{\lambda }\beta < \alpha \le \frac{\lambda }{\lambda -1} \beta$. Using (3.1), we compute

$$\begin{aligned} \omega _{2} = \frac{\pi ^{2}}{6}, \quad \omega _{1} = -\frac{1}{2}, \quad L_{f_3}(0) = \frac{1}{24}, \quad L'_{f_3}(0) = \frac{\log (2\pi )}{24} - \frac{\zeta '(-1)}{2}. \end{aligned}$$

Thus

3.2 $$\begin{aligned} C = \frac{e^{L'_{g_3}(0)}\zeta (3)^{\frac{11}{72}}}{\sqrt{2} \cdot 3^{\frac{47}{72}} \cdot \pi ^{\frac{7}{36}}}, \quad b = \frac{47}{72}, \quad A_1 = \frac{(3\pi )^{\frac{2}{3}} \zeta (3)^{\frac{1}{3}}}{2}, \quad A_2 = - \frac{\pi ^{\frac{4}{3}}}{4 \cdot 3^{\frac{2}{3}} \cdot \zeta (3)^{\frac{1}{3}}}. \end{aligned}$$

Using that $c_{2} = -\frac{\pi ^{2}}{12}$ and $c_{1} = \frac{\pi ^{2}\zeta (3)}{3}$ we next evaluate

$$\begin{aligned} A_3&= -\frac{\pi ^2}{288 \zeta (3)}. \end{aligned}$$

Finally, we find with Theorem 2.2 and (3.2)

$$\begin{aligned} N_3(n)&\sim \frac{e^{-\frac{\zeta '(-1)}{2} -\frac{\pi ^2}{288 \zeta (3)}}\zeta (3)^{\frac{11}{72}}}{2^{\frac{11}{24}} \cdot 3^{\frac{47}{72}} \cdot \pi ^{\frac{11}{72}} \cdot n^{\frac{47}{72}}} \exp \left( \frac{(3\pi )^{\frac{2}{3}} \zeta (3)^{\frac{1}{3}}}{2} n^{\frac{2}{3}} - \frac{\pi ^{\frac{4}{3}}}{4 \cdot 3^{\frac{2}{3}} \cdot \zeta (3)^{\frac{1}{3}}} n^{\frac{1}{3}} \right) . \end{aligned}$$

With Lemma 3.1 and Theorem 2.1 we conclude that the exponents in the polynomial terms in the expansions of $N_3(n)$ are given by $\frac{1}{3} \mathbb {N}_0$, as $(\mathcal {M} + \mathcal {N}) \cap [0,\infty ) = \frac{1}{3} \mathbb {N}_0$. $\square$

### The cases $\ell \in \{4,5\}$

We are now ready to determine the asymptotic behavior of $N_4(n)$ and $N_5(n)$.

#### Theorem 3.3

We have, as $n \rightarrow \infty ,$

$$\begin{aligned} N_{4}(n)&\sim \frac{e^\frac{\zeta '(-2)}{24} \pi ^{\frac{1}{4}} \zeta (3)^{\frac{1}{8}}}{2^{\frac{13}{8}}\cdot 3^{\frac{1}{4}}\cdot 5^{\frac{1}{8}} \cdot n^{\frac{5}{8}}} \exp \left( \frac{2^{\frac{7}{4}} \cdot \pi ^{\frac{3}{2}} \cdot \zeta (3)^{\frac{1}{4}}}{3^{\frac{3}{2}} \cdot 5^{\frac{1}{4}}}n^{\frac{3}{4}} + A_{4,2}n^{\frac{1}{2}} + A_{4,3}n^{\frac{1}{4}} + A_{4,4}\right) \left( 1 + \sum _{j \ge 1} \frac{B_{4,j}}{n^{\frac{j}{4}}}\right) ,\\ N_{5}(n)&\sim \frac{e^{\frac{\zeta '(-2)}{2880}}(\pi \zeta (3) \zeta (5))^{\frac{1}{10}}}{2^{\frac{2}{5}} \cdot 3^{\frac{1}{5}} \cdot 5^{\frac{3}{5}} \cdot n^{\frac{3}{5}}} \\&\quad \times \exp \left( \frac{5^{\frac{4}{5}} \cdot \pi ^{\frac{6}{5}} \cdot (\zeta (3) \zeta (5))^{\frac{1}{5}}}{2^{\frac{9}{5}} \cdot 3^{\frac{2}{5}}}n^{\frac{4}{5}} + A_{5,2}n^{\frac{3}{5}} + A_{5,3}n^{\frac{2}{5}} + A_{5,4}n^{\frac{1}{5}} + A_{5,5}\right) \left( 1 + \sum _{j \ge 1} \frac{B_{5,j}}{n^{\frac{j}{5}}}\right) , \end{aligned}$$

with computable constants $A_{4,j}$ $(2 \le j \le 4)$ and $A_{5,j}$ $(2 \le j \le 5)$ and certain $B_{4,j}$ and $B_{5,j}$.

#### Proof

By Theorem 2.1 we have, for $\ell \in \{4,5\}$, as $\alpha = \ell -1$, $L_{f_\ell }(0) = 0$, and $\omega _{\ell -1} = \zeta (2) \zeta (3) \cdots \zeta (\ell -1)$ (all by Proposition 2.7)

$$\begin{aligned} C = \frac{e^{L_{f_\ell }'(0)} (\ell -1)!^{\frac{1}{2\ell }}\sqrt{Z_\ell }}{\sqrt{2\pi \ell }}, \quad b = \frac{\ell +1}{2\ell }. \end{aligned}$$

We find

$$\begin{aligned} L'_{f_4}(0) = \frac{\zeta '(-2)}{24}, \quad L'_{g_5}(0) = \frac{\zeta '(-2)}{2880}. \end{aligned}$$

The constants $A_{4,1}$ and $A_{5,1}$ can now be computed by straightforward calculations. With Lemma 3.1 and Theorem 2.1 we conclude that the exponents in the polynomial terms in the expansions of $N_\ell (n)$ are given by $\frac{1}{\ell } \mathbb {N}_0$ (for $\ell \in \{4,5\}$), as $(\mathcal {M} + \mathcal {N}) \cap [0,\infty ) = \frac{1}{\ell } \mathbb {N}_0$. $\square$

### Proof of Theorem 1.2

To prove Theorem 1.2, we do some preliminary considerations. By Lemma 3.2 of [8] we have that, as $z \rightarrow 0$ in a cone in the right half plane,

3.3 $$\begin{aligned} \Phi _{f_\ell }(z) = \sum _{\begin{array}{c} \nu \in -\mathcal {P}_R\setminus \{0\} \end{array}} \textrm{Res}_{s=-\nu } L_{f_\ell }^*(s)z^{\nu } - L_{f_\ell }(0){\text {Log}}(z) + L_{f_\ell }'(0) + O_{R}\left( |z|^R\right) \end{aligned}$$

and for $$k\in \mathbb {N}$$

3.4 $$\begin{aligned} \Phi _{f_\ell }^{(k)}(z) = \sum _{\begin{array}{c} \nu \in -\mathcal {P}_R\setminus \{0\} \end{array}} (\nu )_k \textrm{Res}_{s=-\nu } L_{f_\ell }^*(s)z^{\nu - k} + \frac{(-1)^k(k-1)! L_{f_\ell }(0)}{z^k} + O_{R,k}\left( |z|^{R-k}\right) , \end{aligned}$$

where $(s)_k:= s(s-1) \cdots (s-k+1)$. By Proposition 2.7, $L_{f_\ell }$ has exactly three positive poles $\{\ell -3, \ell -2, \ell -1\}$. As $L_{f_\ell }(0) = 0$ by Proposition 2.7 the above becomes

3.5 $$\begin{aligned} - \Phi '_{f_\ell }(z) = \frac{C_1}{z^\ell } + \frac{C_2}{z^{\ell -1}} + \frac{C_3}{z^{\ell -2}} + O_{R}\left( |z|^{R-1}\right) , \end{aligned}$$

where we have, according to Proposition 2.7 and $k=1$ in (3.4),

3.6 $$\begin{aligned}&C_1 = (\ell -1)! \zeta (\ell )\prod _{\begin{array}{c} 0\le k\le \ell -2 \\ k \not = \ell -2 \end{array}} \zeta \left( \ell -1 - k\right) > 0, \quad C_2 = (\ell -2)! \zeta (\ell -1)\prod _{\begin{array}{c} 0\le k\le \ell -2 \\ k \not = \ell -3 \end{array}} \zeta (\ell -2 - k) \ne 0,\nonumber \\&C_3 = (\ell -3)! \zeta (\ell -2)\prod _{\begin{array}{c} 0\le k\le \ell -2 \\ k \not = \ell -4 \end{array}} \zeta (\ell -3- k) \ne 0. \end{aligned}$$

To calculate the exponent sets $\mathcal {L}, \mathcal {M},$ and $\mathcal {N}$, we apply Proposition 2.3. Let $\alpha := \ell -1$. By Proposition 2.3 the exponents of the saddle point function $\varrho _{\ell ,n}$ belonging to $f_\ell$ lie in $\frac{1}{\ell } \mathbb {N}$. Our next goal is to prove the following version of Lemma 4.3 of [8].

#### Lemma 3.4

For $\ell \ge 4,$ let $f:= f_{\ell }$. Then, as $n\rightarrow \infty ,$

$$\begin{aligned} \varrho _{\ell ,n} \sim \sum _{j \ge 1} \frac{K_{\ell ,j}}{n^{\frac{j}{\ell }}} \end{aligned}$$

for some constants $K_{\ell ,j}$ independent of *n* that can be calculated explicitly using $a_k(x),$ $b_k(x),$ and $e_h$ defined below in (3.10), (3.13), and (3.14), respectively. We have

3.7 $$\begin{aligned} K_{\ell ,1} = C_1^{\frac{1}{\ell }}. \end{aligned}$$

#### Proof

Note that it suffices to treat all of the following asymptotic expansions formally, as in Sect. 3 of [8] it was shown that the analytical considerations can be made rigorous under the assumptions of Theorem 2.1.

By Proposition 2.3, the exponents of $\varrho _{\ell ,n}$ lie in $\frac{1}{\ell }\mathbb {N}$. Hence, by Proposition 3.3 of [8] there exist $K_{\ell ,j} \in \mathbb {R}$, such that

$$\begin{aligned} \varrho _{\ell ,n} \sim \sum _{j \ge 1} \frac{K_{\ell ,j}}{n^{\frac{j}{\ell }}} \quad (n \rightarrow \infty ). \end{aligned}$$

With (3.5) we obtain $a_{-\Phi '_{f_\ell },1} = C_1$. We also have $a_{\varrho _\ell ,1} = a_{-\Phi '_{f_\ell },1}^{\frac{1}{\ell }}$ by Proposition 2.3, since $\alpha +1 = \ell$. We conclude that $K_{\ell ,1} = a_{\varrho _\ell ,1} = C_1^{\frac{1}{\ell }}$.

For the exact values of the coefficients $K_{\ell ,j}$ we need to resolve

3.8 $$\begin{aligned} \frac{C_1}{\varrho _{\ell ,n}^{\ell }} + \frac{C_2}{\varrho _{\ell ,n}^{\ell -1}} + \frac{C_3}{\varrho _{\ell ,n}^{\ell -2}} - n = 0 \end{aligned}$$

by (3.5) and Proposition 2.3. In order to do so, we divide (3.8) by *n* to obtain

$$\begin{aligned} C_1 \left( \frac{n^{-\frac{1}{\ell }}}{\varrho _{\ell ,n}}\right) ^\ell + C_2 n^{-\frac{1}{\ell }} \left( \frac{n^{-\frac{1}{\ell }}}{\varrho _{\ell ,n}}\right) ^{\ell -1} + C_3 n^{-\frac{2}{\ell }} \left( \frac{n^{-\frac{1}{\ell }}}{\varrho _{\ell ,n}}\right) ^{\ell -2} - 1 = 0. \end{aligned}$$

Thus we are interested in points $(x,z) = (x,z(x))$ on the curve

$$\begin{aligned} C_1 z^\ell + C_2 x z^{\ell -1} + C_3 x^2 z^{\ell -2} -1 = 0 \end{aligned}$$

for small values of *x*, where $z = \frac{n^{-\frac{1}{\ell }}}{\varrho _{\ell ,n}}$. Making the change of variables

3.9 $$\begin{aligned} z =: w + C_1^{-\frac{1}{\ell }} \end{aligned}$$

we obtain

$$\begin{aligned} C_1 \sum _{k=0}^{\ell } {\ell \atopwithdelims ()k} w^{k} C_1^{-\frac{\ell -k}{\ell }} + C_2 x \sum _{k=0}^{\ell -1} {\ell -1 \atopwithdelims ()k} w^{k} C_1^{-\frac{\ell - 1-k}{\ell }} + C_3 x^2 \sum _{k=0}^{\ell -2} {\ell -2 \atopwithdelims ()k} w^{k} C_1^{-\frac{\ell -2-k}{\ell }} - 1= 0, \end{aligned}$$

using the binomial theorem. We rewrite this in the shape:

$$\begin{aligned} \sum _{k=0}^{\ell } a_k(x) w^k = 0, \end{aligned}$$

where

3.10 $$\begin{aligned} a_k(x) := {\ell \atopwithdelims ()k} C_1^{\frac{k}{\ell }} + C_2 {\ell -1 \atopwithdelims ()k} C_1^{\frac{k+1}{\ell }-1}x + C_3 {\ell -2 \atopwithdelims ()k} C_1^{\frac{k+2}{\ell }-1}x^2 - \delta _{k=0}, \end{aligned}$$

where $\delta _{\mathcal {S}}:=1$ if a statement $\mathcal {S}$ holds and $\delta _{\mathcal {S}}:= 0$ otherwise. In other words, each $a_k(x)$ is a polynomial in *x* of degree at most 2. As

$$a_0(x) = C_2C_1^{\frac{1}{\ell }-1}x + C_3C_1^{\frac{2}{\ell }-1}x^2,$$

we obtain the identity

3.11 $$\begin{aligned} \varphi _x(w) := \sum _{k=1}^{\ell } a_k(x) w^k = -a_0(x) = - C_2C_1^{\frac{1}{\ell }-1}x - C_3C_1^{\frac{2}{\ell }-1}x^2. \end{aligned}$$

We now apply Lemma 2.5 to (3.11) to Taylor approximate its root close to $w=0$ as $x \rightarrow 0$. As $C_1 \not = 0$ (we even show $C_{1}>0$ in (3.6)), we have, by definition

3.12 $$\begin{aligned} a_1(x) = \ell C_1^{\frac{1}{\ell }} + (\ell -1)C_2C_1^{\frac{2}{\ell }-1}x + (\ell -2)C_3C_1^{\frac{3}{\ell }-1}x^2\not = 0 \end{aligned}$$

for all *x* sufficiently small (i.e., all *n* sufficiently large). Thus $\varphi _x(0) = 0$ and $\varphi _x'(0) = a_1(x) \not = 0$. Thus we may apply Lemma 2.5 and obtain that the coefficients of the inverse function are

3.13 $$\begin{aligned}&b_k(x) = \frac{1}{ka_1(x)^k} \sum _{\begin{array}{c} \ell _1,\ell _2, \ell _3 \dots \ge 0 \\ \ell _1 + 2\ell _2 + \cdots = k-1 \end{array}}\quad (-1)^{\ell _1 + \ell _2 +\ell _3+\cdots } \frac{k \cdots (k-1+\ell _1+\ell _2+\cdots )}{\ell _1! \ell _2! \ell _3! \cdots }\nonumber \\&\hspace{5cm}\times \left( \frac{a_2(x)}{a_1(x)}\right) ^{\ell _1} \left( \frac{a_3(x)}{a_1(x)}\right) ^{\ell _2} \cdots \end{aligned}$$

As a result, for *x* sufficiently small, we can solve (3.11) with

3.14 $$\begin{aligned} w(x) = \sum _{k \ge 1} b_k(x) \left( - C_2C_1^{\frac{1}{\ell }-1}x - C_3C_1^{\frac{2}{\ell }-1}x^2\right) ^k =: \sum _{h \ge 0} e_h x^h \end{aligned}$$

with $e_h \in \mathbb {R}$. We now have an expansion for $w = z - C_1^{-\frac{1}{\ell }}$ in powers of $n^{-\frac{1}{\ell }}$. Resolving (3.9) (recall $z = \frac{n^{-\frac{1}{\ell }}}{\varrho _{\ell ,n}}$), i.e.,

$$\begin{aligned} \frac{n^{-\frac{1}{\ell }}}{\varrho _{\ell ,n}} = C_1^{-\frac{1}{\ell }} + w\left( n^{-\frac{1}{\ell }}\right) \end{aligned}$$

leads to the closed expression

$$\begin{aligned} \varrho _{\ell ,n} = \frac{n^{-\frac{1}{\ell }}}{C_1^{-\frac{1}{\ell }} + w\left( n^{-\frac{1}{\ell }}\right) } = C_1^{\frac{1}{\ell }} n^{-\frac{1}{\ell }} \sum _{m \ge 0} C_1^{\frac{m}{\ell }} w\left( n^{-\frac{1}{\ell }}\right) ^m. \end{aligned}$$

The lemma now follows as the previous equation gives the desired expansion. $\square$

We are now ready to prove Theorem 1.2.

#### Proof of Theorem 1.2

We identify the part of the asymptotic in

$$e^{n\varrho _{\ell ,n}}G_{f_\ell }(\varrho _{\ell ,n}) = e^{n\varrho _{\ell ,n} + \Phi _{f_\ell }(\varrho _{\ell ,n})}$$

with non-negative exponents. In other words, we ask for the term

3.15 $$\begin{aligned} e^{n\varrho _{\ell ,n} + \Phi _{f_\ell }(\varrho _{\ell ,n})} = \exp \left( \left[ n\varrho _{\ell ,n} + \Phi _{f_\ell }(\varrho _{\ell ,n})\right] _*\right) (1 + o(1)), \end{aligned}$$

where the notation $[]_*$ means the part of an asymptotic expansion $\sum _{j \ge 1} a_j n^{\beta _j}$ with $\beta _j \ge 0$. First we get with Lemma 3.4, as $n \rightarrow \infty$,

3.16 $$\begin{aligned} n\varrho _{\ell ,n} = \sum _{j=1}^{\ell } K_{\ell ,j}n^{\frac{\ell -j}{\ell }} + O\left( n^{-\frac{1}{\ell }}\right) . \end{aligned}$$

Moreover, with (3.3) and integrating (3.5) we get as $z \rightarrow 0^+$ (in a cone)

$$\begin{aligned} \Phi _{f_\ell }(z) = \frac{C_1}{(\ell -1)z^{\ell -1}} + \frac{C_2}{(\ell -2)z^{\ell -2}} + \frac{C_3}{(\ell -3)z^{\ell -3}} + L'_{g_\ell }(0) + o(1), \end{aligned}$$

since $L_{f_{\ell }}(0)=0$. Thus in particular, as $n \rightarrow \infty$,

3.17 $$\begin{aligned} \Phi _{f_\ell }(\varrho _{\ell ,n}) = \frac{C_1}{(\ell -1) \varrho _{\ell ,n}^{\ell -1}} + \frac{C_2}{(\ell -2) \varrho _{\ell ,n}^{\ell -2}} + \frac{C_3}{(\ell -3)\varrho _{\ell ,n}^{\ell -3}} + L'_{g_\ell }(0) + o(1). \end{aligned}$$

We next use (3.7) and rewrite

$$\begin{aligned} \frac{1}{\varrho _{\ell ,n}}&\sim \frac{1}{C_1^{\frac{1}{\ell }}n^{-\frac{1}{\ell }} + \sum _{j \ge 2} K_{\ell ,j}n^{-\frac{j}{\ell }}} = \frac{n^{\frac{1}{\ell }}}{C_1^{\frac{1}{\ell }}} \left( 1 + \frac{1}{C_1^{\frac{1}{\ell }}} \sum _{j \ge 1} \frac{K_{\ell ,j+1}}{n^{\frac{j}{\ell }}} \right) ^{-1}. \end{aligned}$$

We can work with the following identity regarding formal power series:

$$\begin{aligned} \frac{1}{1 + \sum _{k \ge 1} b_k z^k} = \sum _{m \ge 0} (-1)^m \left( \sum _{k \ge 1} b_k z^k\right) ^m = 1 + \sum _{n \ge 1} c_n z^n, \end{aligned}$$

with

$$\begin{aligned} c_n = \sum _{\begin{array}{c} j_1, j_2, \ldots , j_{n} \ge 0 \\ j_1 + 2j_2 + 3j_3 + \cdots = n \end{array}} (-1)^{j_1+j_2+\cdots } {j_1 + j_2 + j_3 + \cdots \atopwithdelims (){j_1, j_2, j_3, \ldots }} b_1^{j_1} b_2^{j_2} b_3^{j_3}. \end{aligned}$$

We thus obtain

$$\begin{aligned} \frac{1}{\varrho _{\ell ,n}} \sim n^{\frac{1}{\ell }} \sum _{m \ge 0} \frac{D_{m}}{n^{\frac{m}{\ell }}}, \end{aligned}$$

where

$$\begin{aligned} D_{m} := C_1^{-\frac{1}{\ell }}\sum _{\begin{array}{c} j_1, j_2, \ldots \ge 0 \\ j_1 + 2j_2 + 3j_3 + \cdots = m \end{array}} (-1)^{j_1+j_2+\cdots } {j_1 + j_2 + j_3 + \cdots \atopwithdelims (){j_1, j_2, j_3, \ldots }} C_1^{-\frac{m}{\ell }} K_{\ell ,2}^{j_1} K_{\ell ,3}^{j_2} K_{\ell ,4}^{j_3}. \end{aligned}$$

Using the multinomial theorem again, we find

3.18 $$\begin{aligned}&\frac{C_1}{(\ell -1) \varrho _{\ell ,n}^{\ell -1}} + \frac{C_2}{(\ell -2) \varrho _{\ell ,n}^{\ell -2}} + \frac{C_3}{(\ell -3)\varrho _{\ell ,n}^{\ell -3}} \nonumber \\&\quad =\frac{C_1}{\ell -1} n^{\frac{\ell -1}{\ell }} \sum _{k \ge 0} n^{-\frac{k}{\ell }} \sum _{\begin{array}{c} m_1, m_2, \ldots \ge 0 \\ m_1 + m_2 + \cdots = \ell -1 \\ m_2 + 2m_3 + \cdots = k \end{array}} {\ell - 1 \atopwithdelims ()m_1, m_2, \ldots } D_{0}^{m_1} D_{1}^{m_2} \cdots \nonumber \\&\qquad +\frac{C_2}{\ell -2} n^{\frac{\ell -2}{\ell }} \sum _{k \ge 0} n^{-\frac{k}{\ell }} \sum _{\begin{array}{c} m_1, m_2, \ldots \ge 0\\ m_1 + m_2 + \cdots = \ell -2 \\ m_2 + 2m_3 + \cdots = k \end{array}} {\ell - 2 \atopwithdelims ()m_1, m_2, \ldots } D_{0}^{m_1} D_{1}^{m_2} \cdots \nonumber \\&\qquad +\frac{C_3}{\ell -3} n^{\frac{\ell -3}{\ell }} \sum _{k \ge 0} n^{-\frac{k}{\ell }} \sum _{\begin{array}{c} m_1, m_2, \ldots \ge 0\\ m_1 + m_2 + \cdots = \ell -3 \\ m_2 + 2m_3 + \cdots = k \end{array}} {\ell - 3 \atopwithdelims ()m_1, m_2, \ldots } D_{0}^{m_1} D_{1}^{m_2}. \end{aligned}$$

We next determine closed formulas for the exponent coefficients $A_{\ell ,k}$. We first find with Theorem 2.1

3.19 $$\begin{aligned} A_{\ell ,1}&= \left( 1 + \frac{1}{\ell -1}\right) (\zeta (2) \cdots \zeta (\ell -1) (\ell -1)! \zeta (\ell ))^{\frac{1}{\ell }}. \end{aligned}$$

Also note that, combining Theorem 2.1 and Lemma 2.4, we obtain

$$\begin{aligned}&\frac{e^{n\varrho _{\ell ,n}}G_{f_\ell }(\varrho _{\ell ,n})}{\sqrt{2\pi }}\left( \sum \limits _{j=1}^N \frac{d_j }{n^{\nu _j}} + O_{L,R}\left( n^{-\min \left\{ \frac{L+1}{\alpha +1}, \frac{R+\alpha }{\alpha +1}+\frac{\alpha +2}{2(\alpha +1)}\right\} }\right) \right) \\&\quad = \frac{C}{n^b}\exp \left( A_1n^\frac{\alpha }{\alpha +1}+\sum _{j=2}^M A_jn^{\alpha _j}\right) \left( 1+\sum \limits _{j=2}^N \frac{B_j}{n^{\beta _j}} + O_{L,R}\left( n^{-\min \left\{ \frac{2L-\alpha }{2(\alpha +1)},\frac{R}{\alpha +1}\right\} }\right) \right) , \end{aligned}$$

for some numbers $0 \le \alpha _M< \cdots< \alpha _2 < \frac{\alpha }{\alpha +1}$. Consequently with (3.15), comparing the exponential terms with powers in $\mathcal {L} \cap [0,\infty )$ (note that the term $L_{f_\ell }'(0)$ is treated separately, so we have to subtract it),

3.20 $$\begin{aligned} \left[ n\varrho _{\ell ,n} + \Phi _{f_\ell }(\varrho _{\ell ,n}) - L'_{g_\ell }(0)\right] _*&= A_1n^\frac{\alpha }{\alpha +1}+\sum _{j=2}^M A_jn^{\alpha _j} =: \sum _{k=1}^\ell A_{\ell ,k} n^{\frac{\ell -k}{\ell }}. \end{aligned}$$

Using Lemma 3.1 employing (3.16), (3.17), and (3.18), to obtain for $k \ge 2$,

3.21 $$\begin{aligned} A_{\ell ,k}&= K_{\ell ,k} + \frac{C_1}{\ell -1} \hspace{-0.4cm} \sum _{\begin{array}{c} m_1, m_2, \ldots \ge 0 \\ m_1 + m_2 + \cdots = \ell -1 \\ m_2 + 2m_3 + \cdots = k-1 \end{array}} \hspace{-0.4cm} {\ell - 1 \atopwithdelims ()m_1, m_2, \ldots } D_{0}^{m_1} D_{1}^{m_2} \cdots \nonumber \\&\quad + \frac{C_2}{\ell -2} \hspace{-0.4cm} \sum _{\begin{array}{c} m_1, m_2, \ldots \ge 0\\ m_1 + m_2 + \cdots = \ell -2 \\ m_2 + 2m_3 + \cdots = k-2 \end{array}} \hspace{-0.4cm} {\ell - 2 \atopwithdelims ()m_1, m_2, \ldots } D_{0}^{m_1} D_{1}^{m_2} \cdots \nonumber \\&\quad + \frac{C_3}{\ell -3} \hspace{-0.4cm} \sum _{\begin{array}{c} m_1, m_2, \ldots \ge 0\\ m_1 + m_2 + \cdots = \ell -3 \\ m_2 + 2m_3 + \cdots = k-3 \end{array}} \hspace{-0.4cm} {\ell - 3 \atopwithdelims ()m_1, m_2, \ldots } D_{0}^{m_1} D_{1}^{m_2}. \end{aligned}$$

If $\ell \ge 6$, then we have $L'_{f_\ell }(0) = 0$, since $L_{f_\ell }$ has a zero in $s=0$ of order at least 2. This simplifies the formula to the one in Theorem 1.2. Theorem 2.1, Proposition 2.7, and

$$\begin{aligned} C = \frac{(\ell -1)!^{\frac{1}{2\ell }} Z_\ell ^{\frac{1}{2}}}{\sqrt{2\pi \ell }} \end{aligned}$$

as well as (3.19) gives the value of $A_{\ell ,1}$. With Lemma 3.1 and Theorem 2.1 we conclude that the exponents in the polynomial terms in the expansions of $N_\ell (n)$ are given by $\frac{1}{\ell } \mathbb {N}_0$, as $(\mathcal {M} + \mathcal {N}) \cap [0,\infty ) = \frac{1}{\ell } \mathbb {N}_0$. $\square$

## Log-concavity and the proof of Theorem 1.3

### Proof of Theorem 1.3

In this subsection, we prove our general result on log-concavity.

#### Proof of Theorem 1.3

We have

4.1 $$\begin{aligned}&c(n)^2 - c(n+1) c(n-1) \sim C^2\left( \frac{\exp \left( 2\sum _{\lambda \in \mathcal {S}}A_\lambda n^\lambda \right) }{n^{2\kappa }}\left( \sum _{\mu \in \mathcal {T}} \frac{\beta _\mu }{n^\mu }\right) ^2\right. \nonumber \\&\quad \left. -\frac{\exp \left( \sum _{\lambda \in \mathcal {S}}A_\lambda \left( (n+1)^\lambda +(n-1)^\lambda \right) \right) }{(n+1)^\kappa (n-1)^\kappa }\sum _{\mu \in \mathcal {T}}\frac{\beta _\mu }{(n+1)^\mu }\sum _{\nu \in \mathcal {T}}\frac{\beta _\nu }{(n-1)^\nu }\right) . \end{aligned}$$

We now claim that for all $\lambda \in \mathcal {S}$, we have

4.2 $$\begin{aligned} \exp \left( A_\lambda \left( (n+1)^\lambda +(n-1)^\lambda -2n^\lambda \right) \right) = 1 + \frac{\gamma _{\lambda ,1}}{n^{2-\lambda }} + o\left( n^{-2+\lambda }\right) \end{aligned}$$

for $\gamma _{\lambda ,1} \in \mathbb {R}$. To see this, write $\lambda = \frac{a}{b}$ with $\gcd (a,b)=1$ and set $x:=n^{-\frac{1}{b}}$. Then

$$\begin{aligned} (n+1)^\frac{a}{b}+(n-1)^\frac{a}{b}-2n^\frac{a}{b}&= \left( \frac{1}{x^b}+1\right) ^\frac{a}{b} + \left( \frac{1}{x^b}-1\right) ^\frac{a}{b} - 2x^{-a}\\&= \frac{1}{x^a} \left( \left( 1+x^b\right) ^\frac{a}{b}+\left( 1-x^b\right) ^\frac{a}{b}-2\right) =: f_{a,b}(x). \end{aligned}$$

Now, as $$y \rightarrow 0$$,

$$\begin{aligned} g_\lambda (y) := (1+y)^\lambda +(1-y)^\lambda -2 = O\left( y^2\right) . \end{aligned}$$

Thus $f_{a,b}$ has a Taylor expansion of the shape

$$\begin{aligned} f_{a,b}(x) = \sum _{j\ge 1} \alpha _{\lambda ,j} x^{2b j-a}. \end{aligned}$$

Thus

$$\begin{aligned} f_{a,b}\left( n^{-\frac{1}{b}}\right) = \sum _{j\ge 1} \frac{\alpha _{\lambda ,j}}{n^{\frac{2b j-a}{b}}} = \frac{\alpha _{\lambda ,1}}{n^{2-\lambda }}+o\left( n^{-2+\lambda }\right) . \end{aligned}$$

Plugging in the expansion of the exponential function gives the claim, using that $0<\lambda < 1$.

Using (4.2), we obtain

$$\begin{aligned} \exp \left( \sum _{\lambda \in \mathcal {S}}A_\lambda \left( (n+1)^{\lambda }+(n-1)^{\lambda }-2n^\lambda \right) \right)&= \prod _{\lambda \in \mathcal {S}} \left( 1 + \frac{\gamma _{\lambda ,1}}{n^{2-\lambda }} + o\left( n^{-2+\lambda }\right) \right) \\&= 1 + \frac{\gamma _{\lambda ^*,1}}{n^{2-\lambda ^*}} + o\left( n^{-2+\lambda ^*}\right) . \end{aligned}$$

Note that $\gamma _{\lambda ^*,1}=A_{\lambda ^*}\alpha _{\lambda ^*,1}$ and that $\alpha _{\lambda ,1} = \lambda (\lambda -1)<0$.

Next we claim that (for $n > 1$)

4.3 $$\begin{aligned} \frac{1}{(n+1)^\kappa (n-1)^\kappa } =\frac{1}{\left( n^2-1\right) ^\kappa } = n^{-2\kappa } \left( 1+\sum _{j\ge 1}\frac{\delta _j}{n^{2j}}\right) \end{aligned}$$

for certain $\delta _j$. To see this, set $x:=n^{-2}$. Then we want

$$\begin{aligned} \frac{x^{-\kappa }}{\left( \frac{1}{x}-1\right) ^\kappa } = 1 + \sum _{j\ge 1} \delta _j x^j. \end{aligned}$$

The left-hand side is $\frac{1}{(1-x)^\kappa }$ and the claim follows.

Thus, by (4.1),

$$\begin{aligned}&c(n)^2 - c(n+1) c(n-1)\\&\quad \sim C^2 \frac{\exp \left( 2\sum _{\lambda \in \mathcal {S}}A_\lambda n^\lambda \right) }{n^{2\kappa }} \left( \left( \sum _{\mu \in \mathcal {T}}\frac{\beta _\mu }{n^\mu }\right) ^2\right. \\&\qquad \left. -\left( 1+\frac{\gamma _{\lambda ^*,1}}{n^{2-\lambda ^*}} + o\left( n^{-2+\lambda ^*}\right) \right) \left( 1+O\left( n^{-2}\right) \right) \sum _{\mu \in \mathcal {T}} \frac{\beta _\mu }{(n+1)^\mu } \sum _{\nu \in \mathcal {T}} \frac{\beta _\nu }{(n-1)^\nu }\right) . \end{aligned}$$

The sign of this is dictated by

$$\begin{aligned} \sum _{\mu ,\nu \in \mathcal {T}} \frac{\beta _\mu \beta _\nu }{n^{\mu +\nu }} - \left( 1+\frac{\gamma _{\lambda ^*,1}}{n^{2-\lambda ^*}} + o\left( n^{-2+\lambda ^*}\right) \right) \left( 1+O\left( n^{-2}\right) \right) \sum _{\mu \in \mathcal {T}} \frac{\beta _\mu }{(n+1)^\mu } \sum _{\nu \in \mathcal {T}} \frac{\beta _\nu }{(n-1)^\nu }. \end{aligned}$$

As $\beta _0 = 1$, the above equals mod $(o(n^{-2+\lambda ^*}))$,

4.4 $$\begin{aligned}&\sum _{\begin{array}{c} \mu ,\nu \in \mathcal {T}\\ 0\le \mu +\nu \le 2-\lambda ^* \end{array}} \frac{\beta _\mu \beta _\nu }{n^{\mu +\nu }} - \sum _{\begin{array}{c} \mu ,\nu \in \mathcal {T}\\ 0\le \mu +\nu \le 2-\lambda ^* \end{array}} \frac{\beta _\mu \beta _\nu }{(n+1)^\mu (n-1)^\nu } - \frac{\gamma _{\lambda ^*,1}}{n^{2-\lambda ^*}}\nonumber \\&\quad = 2\sum _{\begin{array}{c} \mu \in \mathcal {T}\\ 0\le \mu \le 1-\frac{\lambda ^*}{2} \end{array}} \beta _\mu ^2 \left( \frac{1}{n^{2\mu }}-\frac{1}{(n+1)^\mu (n-1)^\mu }\right) \nonumber \\&\qquad + \!\!\!\!\!\!\!\!\sum _{\begin{array}{c} \mu \in \mathcal {T}\\ 0\le \mu <\nu \\ 1\le \mu +\nu \le 2-\lambda ^* \end{array}} \!\!\!\!\!\!\!\!\!\beta _\mu \beta _\nu \left( \frac{2}{n^{\mu +\nu }}-\frac{1}{(n+1)^\mu (n-1)^\nu }-\frac{1}{(n+1)^\nu (n+1)^\mu }\right) - \frac{\gamma _{\lambda ^*,1}}{n^{2-\lambda ^*}}. \end{aligned}$$

Next, by (4.3) we obtain that (for $n > 1$)

$$\begin{aligned} \frac{1}{(n+1)^\mu (n-1)^\mu } = n^{-2\mu } + O\left( n^{-2\mu -2}\right) . \end{aligned}$$

Similarly, we see that, for certain $\rho _r$,

$$\begin{aligned} \frac{1}{(n+1)^\mu (n-1)^\nu } + \frac{1}{(n+1)^\nu (n-1)^\mu } = 2n^{-\mu -\nu } \left( 1+\sum _{r\ge 1}\frac{\rho _r}{n^{2r}}\right) . \end{aligned}$$

Thus (4.4) becomes

$$\begin{aligned} O\left( n^{-2}\right) - \frac{\gamma _{\lambda ^*,1}}{n^{2-\lambda ^*}}. \end{aligned}$$

To conclude the claim, we note that we have

$$\begin{aligned} \text{ sgn }(-\gamma _{\lambda ^*,1}) = \text{ sgn }(A_{\lambda ^*}) = 1.   \square \end{aligned}$$

### Examples

We can show the following result.

#### Corollary 4.1

Let $f :\mathbb {N}\rightarrow \mathbb {N}_0$ satisfy all conditions of Theorem 2.1, and assume that we can choose *L* in (P1) arbitrarily large. Assume furthermore that $L_f(s)$ has a meromorphic continuation to $\mathbb {C}$ with only rational poles. Then $p_f(n)$ is log-concave for *n* sufficiently large.

#### Proof

Theorem 2.1 provides an expansion for $p_f(n)$ as in Theorem 1.3, since the rational poles of $L_f(s)$ guarantee that all exponents occurring in the expansion of $p_f(n)$ are again rational. Note that $\lambda ^* = \frac{\alpha }{\alpha +1}$ and^9^$A_1 > 0$ again by Theorem 2.1. This gives then the claim. $\square$

There are several further applications. Examples are, besides the classical partitions and plane partitions, partitions into *k*-gonal numbers $p_k(n)$, and the number of *n*-dimensional representations for the groups $\mathfrak {su}(3)$ and $\mathfrak {so}(5)$, denoted by $r_{\mathfrak {su}(3)}(n)$ and $r_{\mathfrak {so}(5)}(n)$, respectively. The asymptotic behavior of the numbers $r_{\mathfrak {su}(3)}(n)$ was first studied by Romik [26] and later refined by two of the authors in [9]. Asymptotic expressions for $r_{\mathfrak {so}(5)}(n)$ and $p_k(n)$ were given in [8]. We directly obtain the following:

#### Corollary 4.2

Let $k \ge 3$. For *n* sufficiently large, the sequences $p_k(n),$ $r_{\mathfrak {su}(3)}(n),$ and $r_{\mathfrak {so}(5)}(n)$ are log-concave.

Finally, we give another example in a slightly different direction. For $d \in \mathbb {N}_0$, let^10^

4.5 $$\begin{aligned} \sum _{n \ge 0} \textrm{p}_d(n)q^n := \prod _{n \ge 1} \left( 1 - q^n\right) ^{-n^d}. \end{aligned}$$

Note that we have $\textrm{p}_0(n) = p(n)$ and $\textrm{p}_1(n) = \textrm{pp}(n)$ is the plane partition function. Note that the corresponding *L*-series is given by

$$\begin{aligned} \sum _{n \ge 1} \frac{n^d}{n^s} = \zeta (s-d). \end{aligned}$$

It is not hard to check that the conditions (P1), (P2), and (P3) are satisfied, and we have

$$\begin{aligned} \mathcal {P}_R \subseteq \{d+1\} \cup \{-1,-2,-3, \ldots \} \end{aligned}$$

for the set of poles $s \not = 0$ of $L_d^*(s):= \Gamma (s)\zeta (s+1)\zeta (s-d)$. Note that $\alpha = d+1$ and

$$\begin{aligned}&b = \frac{1}{2(d+2)} - \frac{\zeta (-d)}{d+2} + \frac{1}{2}, \quad A_1 = \left( 1 + \frac{1}{d+1}\right) \left( (d+1)! \zeta (d+2) \right) ^{\frac{1}{d+2}}, \\&C = \frac{e^{\zeta '(-d)}((d+1)!\zeta (d+2))^\frac{\frac{1}{2}-\zeta (-d)}{d+2}}{\sqrt{2\pi (d+2)}}. \end{aligned}$$

As a consequence, we have by Theorem 2.1

$$\begin{aligned} \textrm{p}_d(n) \sim \frac{C}{n^b} e^{A_1n^{\frac{d+1}{d+2}}} \left( 1 + \sum _{j \ge 1} \frac{E_{d,j}}{n^{\frac{j}{d+2}}}\right) \end{aligned}$$

with certain $E_{d,j}$. Note that, by the same arguments used for the other examples above, $\textrm{p}_d(n)$ is log-concave for *n* sufficiently large. In [17] it had been proven that in the case of plane partitions, $p_1(n)$ is log-concave for almost all *n* and conjectured that this is already valid for $n \ge 12$. This conjecture had been proven by Ono et al. [25].

## Proof of Theorem 1.5

The idea of the following proof is similar to that of Theorem 1.3.

### Proof of Theorem 1.5

Assume that *a*, $b\gg 1$. Then

$$\begin{aligned}&c(a) c(b) - c(a+b) \sim \frac{C^2}{a^\kappa b^\kappa } \exp \left( \sum _{\lambda \in \mathcal {S}} A_\lambda \left( a^\lambda +b^\lambda \right) \right) - \frac{C}{(a+b)^\kappa } \exp \left( \sum _{\lambda \in \mathcal {S}} A_\lambda (a+b)^\lambda \right) \\&\quad = \frac{C^2}{a^\kappa b^\kappa } \exp \left( \sum _{\lambda \in \mathcal {S}} A_\lambda \left( a^\lambda +b^\lambda \right) \right) \left( 1-\frac{a^\kappa b^\kappa }{C(a+b)^\kappa }\exp \left( \sum _{\lambda \in \mathcal {S}} A_\lambda \left( (a+b)^\lambda -a^\lambda -b^\lambda \right) \right) \right) . \end{aligned}$$

Without loss of generality we may assume that $a\ge b$ and write $a=xb$, $x\ge 1$. Then

$$\begin{aligned}&\frac{a^\kappa b^\kappa }{C(a+b)^\kappa }\exp \left( \sum _{\lambda \in \mathcal {S}} A_\lambda \left( (a+b)^\lambda -a^\lambda -b^\lambda \right) \right) \\&\quad = \frac{x^\kappa b^\kappa }{C(x+1)^\kappa }\exp \left( \sum _{\lambda \in \mathcal {S}} A_\lambda b^\lambda \left( (1+x)^\lambda -x^\lambda -1\right) \right) . \end{aligned}$$

Let $f_\lambda (x):=(1+x)^\lambda -x^\lambda -1$. It is not hard to see that $f'_\lambda (x) < 0$. As $f_\lambda (0)=0$, we have $f_\lambda (x)<0$ for $x\ge 1$. Moreover , using l’Hospital,

$$\begin{aligned} \lim _{x\rightarrow \infty } \left( (x+1)^\lambda -x^\lambda -1\right) = -1. \end{aligned}$$

We conclude $-1 \le f_\lambda (x) < 0$ for $x \ge 1$. This gives the claim. $\square$

## Open questions

We leave some open questions to the interested reader.

1. What can be said about Turán type inequalities for the sequences studied? Note that the case $\ell =2$ of the partition function was treated in ([15], Theorems 1 and 2) by showing that certain classes of Jensen polynomials have real roots. The more general case $\ell \ge 3$ will be much harder.
2. It would be interesting to make the results of this paper (in particular log-concavity) explicit. The hard part would be to make [8] explicit. For example, the case $\ell =2$ of the partition function was treated in ([13], Theorem 1.1). Note that this turns out to be much easier than the higher cases of $\ell$ as in the partition case one has an exact formula, which yields inequalities for the partition function. For some exceptional sets for the log-concavity of $N_\ell (n)$ we refer to Tables 1 and 2 of [3].
3. According to a conjecture by Chen (see Conjecture 1.2 in [13]), the sequence $|C_{2,n}| = n! p(n)$ is log-convex for all $n > 1$, which was shown by DeSalvo and Pak ([13], Theorem 4.1). The question now is whether this is also true for sufficiently large *n* for the $|C_{\ell ,n}|$ for all $\ell \ge 3$, and to what extent this could be related to the results of this work.

## Data Availability

No datasets were generated or analysed during the current study.

## Footnotes

1 The result of Bryan–Fulman had been strongly influenced by Stanley (see [11] the acknowledgments).

2 Further proofs have been obtained by [2, 29]. The work of Bryan and Fulman is a generalization of a combinatorial formulae due to Macdonald [21] and Hirzebruch and Höfer [18].

3 We refer to the work of Lubotzky and Segal [20] for more background on the group theoretic interpretation.

4 Here and in the following such series are meant as asymptotic expansions.

5 For the definition, more background and applications in combinatorics see Stanley [28] and Sect. 5 of Brändén [7].

6 The result can probably also be extended to real exponents, but this case is not required for any of our applications and thus we do not allow this case here.

7 Note that the case $\ell =1$ is trivially true.

8 We go back and forth between functions and sequences in our notations here. For more details see Sect. 2.2 in [8].

9 Note the abuse of notation.

10 See [16], where a generalization of generating function for the partition and plane partition function was studied.
